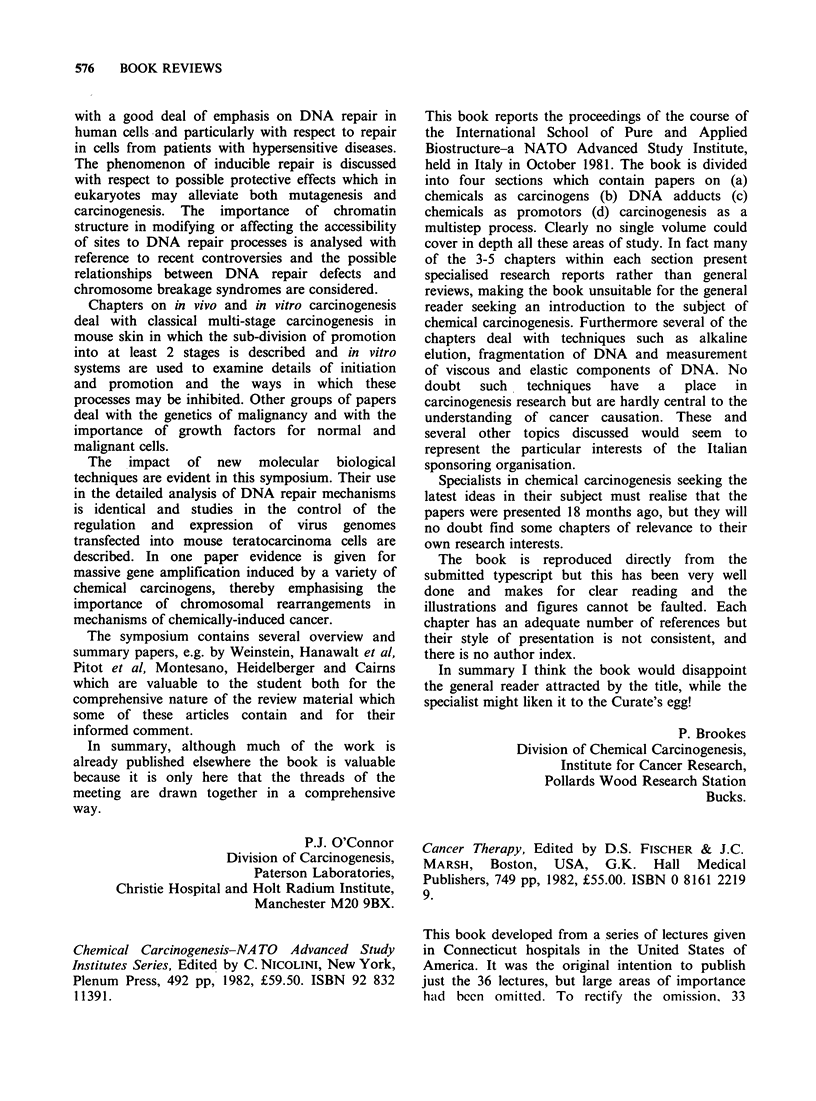# Chemical Carcinogenesis-NATO Advanced Study Institutes Series

**Published:** 1983-04

**Authors:** P. Brookes


*Chemical Carcinogenesis–NATO Advanced Study Institutes Series*, Edited by C. Nicolini, New York, Plenum Press, 492 pp, 1982, £59.50. ISBN 92 832 11391.

This book reports the proceedings of the course of the International School of Pure and Applied Biostructure–a NATO Advanced Study Institute, held in Italy in October 1981. The book is divided into four sections which contain papers on (a) chemicals as carcinogens (b) DNA adducts (c) chemicals as promotors (d) carcinogenesis as a multistep process. Clearly no single volume could cover in depth all these areas of study. In fact many of the 3-5 chapters within each section present specialised research reports rather than general reviews, making the book unsuitable for the general reader seeking an introduction to the subject of chemical carcinogenesis. Furthermore several of the chapters deal with techniques such as alkaline elution, fragmentation of DNA and measurement of viscous and elastic components of DNA. No doubt such techniques have a place in carcinogenesis research but are hardly central to the understanding of cancer causation. These and several other topics discussed would seem to represent the particular interests of the Italian sponsoring organisation.

Specialists in chemical carcinogenesis seeking the latest ideas in their subject must realise that the papers were presented 18 months ago, but they will no doubt find some chapters of relevance to their own research interests.

The book is reproduced directly from the submitted typescript but this has been very well done and makes for clear reading and the illustrations and figures cannot be faulted. Each chapter has an adequate number of references but their style of presentation is not consistent, and there is no author index.

In summary I think the book would disappoint the general reader attracted by the title, while the specialist might liken it to the Curate's egg!

P. Brookes
Division of Chemical Carcinogenesis,
Institute for Cancer Research,
Pollards Wood Research Station
Bucks.